# Microbial communities and inflammatory response in the endometrium differ between normal and metritic dairy cows at 5–10 days post-partum

**DOI:** 10.1186/s13567-018-0570-6

**Published:** 2018-08-02

**Authors:** Ron Sicsic, Tamir Goshen, Rahul Dutta, Noa Kedem-Vaanunu, Veronica Kaplan-Shabtai, Zohar Pasternak, Yuval Gottlieb, Nahum Y. Shpigel, Tal Raz

**Affiliations:** 1 Koret School of Veterinary Medicine, Robert H. Smith Faculty of Agriculture, Food and Environment, The Hebrew University of Jerusalem, Rehovot, Israel; 2 Hachaklait, Mutual Society for Veterinary Services, Caesarea Industrial Park, Caesarea, Israel; 3 Department of Plant Pathology and Microbiology, Robert H. Smith Faculty of Agriculture, Food and Environment, The Hebrew University of Jerusalem, Rehovot, Israel

## Abstract

Post-partum metritis is among the most prevalent disease in dairy cows affecting animal welfare and inflicting considerable economic loses. While post-partum contamination of the uterus is rife in dairy cows, only a fraction of these animals will develop metritis. Our main objective was to compare the bacterial communities and the inflammatory response in the endometrium of healthy and metritic dairy cows. Holstein–Friesian cows (*n* = 35) were sampled immediately following clinical classification as healthy (*n* = 21), suffering from metritis (*n* = 13) or septic metritis (*n* = 1), based on veterinary examination at 5–10 days post-partum. Polymorphonuclear cells (PMN) percentage in endometrial cytology was significantly higher in cows with metritis. Full-thickness uterine biopsy analysis revealed that the luminal epithelium in inter-caruncle areas was preserved in healthy cows, but in metritis it was compromised, with marked PMN infiltration particularly in the apical endometrium. Gram staining revealed that bacterial load and spatial distribution was associated with disease severity. 16S-rDNA bacterial community analysis revealed unique endometrial bacterial community composition in metritic cows, as compared to more diverse communities among healthy cows. The most abundant phyla in healthy cows were Proteobacteria (31.8 ± 9.3%), Firmicutes (27.9 ± 8.4%) and Bacteroidetes (19.7 ± 7.2%), while Bacteroidetes (60.3 ± 10.3%), Fusobacteria (13.4 ± 5.9%) and Firmicutes (10.5 ± 3.3%) were most abundant in the endometrial mucosa of metritic cows. Relative abundance of Bacteroidetes (19.7 ± 7.2% vs. 60.3 ± 10.3%), Fusobacteria (7.5 ± 5.2% vs. 13.4 ± 5.9%) and Proteobacteria (31.8 ± 9.3% vs. 7.3 ± 5.6%) phyla differed significantly between healthy and metritic cows. In summary, endometrial PMN abundance, spatial distribution and bacterial communities differed between healthy and metritic dairy cows at early post-partum.

**Electronic supplementary material:**

The online version of this article (10.1186/s13567-018-0570-6) contains supplementary material, which is available to authorized users.

## Introduction

Post-partum uterine inflammatory diseases are among the most prevalent diseases in bovine dairy herds worldwide, resulting in major economic losses, mainly through decreased reproductive performance and milk production of affected cows [1–3]. After parturition the uterine endometrium undergoes an involution process, which is characterized by massive tissue remodeling; myometrium contractions, shrinkage of the uterus, sloughing of the caruncles and endometrial regeneration [4]. The presence of epithelial tissue debris and fluid during this period makes the uterine environment favorable for bacterial growth [5]. According to an earlier report [6], nearly 100% of dairy cows are affected by bacterial contamination of the uterus at the first 2–3 weeks post-partum, which may often lead to uterine infection and disease. Although the immune system progressively clears most of the bacterial load, 25–40% of animals may have clinical metritis in the first 2 weeks after calving, and the disease may persist as subclinical endometritis in up to 20–30% of animals [7, 8].

The pathophysiology of uterine diseases in post-partum dairy cows is not completely clear. It is not well understood how in some animals the essential inflammatory process for normal uterine involution develops into metritis and endometritis. This could possibly be affected by a combination of certain microbes that enter the post-partum uterus or are present in the uterus shortly after or even during pregnancy [9, 10], as well as by the defense mechanism and the immune reaction of the cow [6, 11, 12]. The severity of clinical manifestation may depend on the type of bacterial population. Culture-dependent microbiological analysis has revealed the presence of several microbes in the uterus of post-partum metritic cows, such as *Escherichia coli*,* Trueperella pyogenes*, *Fusobacterium necrophorum*, *Prevotella melaninogenica*, *Bacteroides* spp., *Pseudomonas* spp., *Streptococcus* spp., and *Staphylococcus* spp. [13]. However, in recent years, the advancement of culture independent studies of the bacterial communities of post-partum bovine uteri using various techniques, such as DGGE profiling [14, 15], T-RFLP [16], FTIR spectroscopy [17], clone library sequencing [14, 16, 18], and next generation sequencing of 16S-rDNA amplicon libraries [15, 19–23], has led to different results than culture based studies, and has made it possible to identify some bacteria that are difficult to grow in routine classical cultures. Since the analysis in culture independent studies relies on DNA isolated from the sample, it is probable that not all identified bacteria in a sample are viable. Nevertheless, culture independent methods are a powerful tool, and knowing the differences in diversity of the uterine microbiota between healthy and metritic cows may provide additional insight into the bacterial communities related to uterine disorders and the pathophysiology of these reproductive diseases. This knowledge is critical for the development of efficient prevention and therapeutic intervention.

It is well documented that periparturient dairy cows undergo a transition period, in which they may have diminished immune competence due to the interplay of multiple factors like metabolic changes, oxidative stress, nutritional imbalances, and stress due to pain during prolonged parturition and lactation; and therefore, the effectivity of the immune response against microbial challenges may be altered [24–26]. Polymorphonuclear cells (PMN) are the first and most significant inflammatory cells to be recruited from the peripheral blood circulation into the endometrium and uterine lumen as part of the post-partum uterine immune response to microbes [7]. Therefore, a higher percentage of PMN is usually found in cows suffering from uterine inflammatory diseases in comparison to healthy cows. However, the normal phagocytic activity of the neutrophils may be compromised by endocrine and metabolic changes around the time of parturition [27]. This impairment of the ability of neutrophils to respond to infectious challenges in periparturient dairy cows may potentially contribute to the high incidence of metritis in early lactation [28, 29]. However, there is a lack of information regarding the distribution and activity of the PMN in affected uterine tissue sections.

Numerous studies have reported endometrial cytological analysis from the fourth week post-partum onward. Endometrial cytological analysis of the first 2 weeks post-partum is usually not performed, in part because of technical difficulties (large, thin walled and abdominal uterus), but mainly because the clinical diagnosis of uterine disease at this time (metritis) relies on obvious clinical signs, such as enlarged uterus and mucopurulent fetid vaginal discharge [30, 31]. Histopathological studies of full thickness biopsies of the bovine uterus in the first 2 weeks post-partum are indeed available [32–34], but unfortunately those studies are limited by a lack of specimens suffering from metritis, no description of PMN spatial distribution, and the fact that most of the samples were obtained by necropsy.

The objective of this study was to compare healthy Holstein–Friesian dairy cows to cows diagnosed with metritis in the early lactation (5–10 days in milk; DIM), in regard to uterine cytology (endometrial swabs), histology (full thickness uterine biopsies), bacterial communities (16S-rDNA pyrosequencing analysis of swab and biopsy samples) and reproductive performance.

## Materials and methods

### Animals and study design

All animal procedures were performed by licensed veterinarians with consent of the animal owner and approved by the Agricultural Research Organization Animal Care Committee for the Hachaklait, Mutual Society for Veterinary Services (Approval No. IL-233/10). Post-partum Holstein–Friesian cows (*n* = 35) of different parities (1–6) were included in this cross-sectional study. Cows were housed in loose housing systems in large, completely covered open sheds and fed a total mixed ration according to the recommendations of the Nutrient Requirement of Dairy Cattle. Cows were milked three times daily in a computerized milking parlor, with an average annual milk yield of 11 500 kg per cow, corrected to 305-days of lactation. Included cows had no history of retained fetal membranes, post-parturient hypocalcemia or mastitis in the current lactation, and did not receive antibiotic or anti-inflammatory treatments prior to sampling. Examination and sampling were performed at 5–10 DIM by the same veterinary practitioner.

Cows were classified as healthy or suffering from metritis as described previously [6, 31]. Briefly, clinical metritis diagnosis was based on the presence of foul smelling, red-brown watery to viscous purulent vaginal discharge, trans-rectal palpation of a thin walled, large uterus containing large amounts of fluid or gas, and the absence of systemic clinical signs such as pyrexia, anorexia or reduction in milk production. Cows with the clinical signs described above and systemic clinical signs (such as fever, anorexia, or depression) were classified as suffering from septic metritis (one cow that was used for full thickness biopsy sampling, as described below). Cows that did not present any of these clinical findings (uterus size and thickness expected for 5–10 DIM, a watery red vaginal discharge without foul smell, and no systemic clinical signs) were classified as healthy. Full individual cow health and reproduction data during lactation were collected from the herd management software (NOA, Israeli Cattle Breeders Association, Caesarea, Israel).

### Endometrial cytology and DNA sampling

Endometrial cytology sampling was performed at 5–10 DIM by the same veterinary practitioner from 30 cows. Prior to sampling, the vulva was washed with a clean brush and soap water, followed by wipes containing 2-propanol and chlorhexidine-gluconate. After placing a clean plastic sleeve on the arm used for sampling, the vulva was lubricated with a sterile lubricant and a sterile double guarded endometrial culture swab (Equi-Vet, Denmark, Cat. No. 290955) was placed through the vagina at the external os of the cervix. Upon retraction of the hand from the vagina, vaginal discharge was evaluated for appearance and volume of secretions and for foul odor, as part of the clinical examination. The same hand was then inserted rectally, and the uterus was palpated to evaluate size, content, thickness of the uterine wall and the degree of involution. The swab was then guided through the cervix into the base of the right uterine horn on the medial side. The tip of the swab was extracted from the protective sheaths, pressed to the endometrium and rotated 5–6 times. The tip was retracted into the protective sheaths, and the swab was taken out of the vagina. The process was repeated with another swab after re-cleaning the vulva and changing the sleeve. Two swabs were successfully obtained from all cows. The tips of the swabs were broken at the indentation on the inner sheath, sealed with the sterile supplied caps, and transferred within 1 h at 4 °C to the laboratory. The first swab was used for DNA extraction and 16S-rDNA bacterial community analysis and the second swab was used for cytological analysis.

The cytology swab was placed in a sterile 15 mL Polypropylene tube containing 1 mL of sterile phosphate buffered saline (PBS). The liquid immersed swab was mixed by vortex at 3000 RPM for 10 s to assist biological matter transfer to the solution. Aliquots of 800 µL of the resultant suspension were loaded onto glass slides by a Cytospin™ centrifuge (Cytospin™ 3, Thermo-Shandon, Waltham, MA USA). Slides were stained with Diff-Quick, and were evaluated by a single experienced observer, who was blinded to the origin of the slides and the status of the cow. Cytological evaluation was done at a magnification of 400× (Nikon Eclipse E400, Tokyo, Japan). For each Diff-Quick stained slide, 100 cells per field (excluding red blood cells) were counted in 10 different fields, and used to calculate PMN percentage in the sample.

### Full thickness uterine biopsy sampling

Full thickness biopsies (from serosa to mucosa) were obtained from five cows by the same veterinary practitioner. Standing right flank laparotomy was performed to obtain biopsies from four of these cows at 7–9 DIM: two cows diagnosed with metritis and two were defined as healthy according to the criteria described above. A fifth biopsy was obtained by necropsy (< 10 min post-mortem) of a cow that died at 7 DIM as a result of per-acute septic metritis. Prior to laparotomy, cows were sedated using 0.05 mg/kg intravenous Xylazine. Aseptic preparation and paravertebral anesthesia were performed at the area of intended incision (T_13_–L_3_). The skin, subcutis and abdominal muscles were incised and bluntly dissected to create a modified grid approach to the abdomen as previously described [35]. The right uterine horn was identified and exteriorized through the incision. A 10 cm long and 5 cm wide spindle shaped full biopsy (from serosa to mucosa), containing a caruncle at mid-length, was taken from the greater curvature using a fresh sterile scalpel. The uterine horn, muscles and subcutis were sutured with 0-USP chromic cat-gut suture material. The skin was sutured using 0-USP nylon. Post-operative care included intramuscular oxytocin (50 IU; twice daily for 3 days), Penicillin-Procaine (20 000 IU/kg once daily for 5 days) and Gentamycin (3 mg/kg once daily for 5 days). Later, three of the four cows had uncomplicated pregnancies and deliveries following artificial insemination (AI). The fourth suffered from perimetritis following surgery, and subsequently was not inseminated. The biopsy from the fifth cow was taken during necropsy from the same uterine location as described above.

The spindle shaped biopsies were transferred to a sterile area and handled with new sterile latex gloves and equipment. Each spindle biopsy was divided to three segments; one containing the caruncle and two intercaruncular segments. Each segment of the biopsy was subdivided into two sub-segments. One sub-segment was placed in a sterile 50 mL polypropylene tube containing 30 mL of 4% para-formaldehyde solution, and transferred to the laboratory for paraffin embedding. The other sub-segment was flash frozen in liquid nitrogen, and stored at −80 °C until DNA extraction and 16S-rDNA bacterial community analysis.

### Total DNA extraction and sequencing

The DNA swabs (*n* = 30) were mixed with sterile PBS as described above and the samples were kept in −80 °C until DNA extraction. Biopsy samples (one caruncular and one intercaruncular sample per cow; *n* = 5 cows, 10 biopsies) were thawed and separately homogenized according to the extraction kit instructions. To avoid bias toward extraction of DNA from Gram negative bacteria, 2 mg of lysozyme (Thermo Fisher Scientific, Cat. No. 89833) were added to each sample and incubated at 37 °C for 1 h. Total DNA was extracted from swabs and biopsy samples using AccuPrep^®^ Genomic DNA Extraction Kit (BIONEER, Republic of Korea, Cat. No. k-3032) following the manufacturer’s protocol.

Total DNA was used for 16S-rDNA bacterial tag encoded FLX amplicon pyrosequencing using the Roche 454 sequencing system as described previously [36]. The primers used for amplification prior to sequencing were 27F (5′ GAG TTT GAT CNT GGC TCA G 3′) and 519R (5′ GTN TTA CNG CGG CKG CTG 3′), resulting in amplicons containing the V1–V3 region of the 16S-rRNA bacterial genes.

## 16S-rDNA bacterial community analysis

The 16S-rRNA gene sequence fasta files (the standard text based format for representing nucleotide sequence [37, 38]) and quality data were extracted from the SFF files generated by the 454 Titanium sequencer (MR DNA, Shallowater, TX USA). Average read length before trimming and quality control was 405 bp. Sequences were grouped according to barcode (1 mismatch allowed) and primer (2 mismatches allowed). Trimming, denoising and quality control were performed using MOTHUR v1.24 [39] according to the standard MOTHUR 454 protocol [40]. Sequences were aligned to the SILVA reference alignment database [39], and filtered to achieve perfect overlap while avoiding base pair overhang. Sequences were pre-clustered using the algorithm described by Huse et al. [41]. Chimeric reads were removed by execution of the UCHIME method using the MOTHUR software [42]. All the non-bacteria reads (chloroplast, mitochondria and kingdom level unclassified reads) were deleted from further analyses.

After cleaning and quality control, 96 988 sequences with an average read length of 242 bp were left. Sequences with >97% similarity were clustered into operational taxonomic units (OTUs) after calculation of pairwise distances between all reads. Phylotyping was based on current RDP-II taxonomy [43]. A data matrix of the OTUs was constructed—a separate column for each OTU and a separate row for each sample. Following relativization of the number of OTUs in a sample to the total number of 454 reads obtained from the same sample, a data point in the matrix corresponds to the abundance of a particular OTU in that sample. No rarefaction was performed for read abundance data following McMurdie and Holmes [44].

### Histopathology analysis

Uterine biopsies were fixed immediately after collection in 4% para-formaldehyde solution for 24 h, and later were dehydrated and paraffin embedded using routine procedure. Sections of 4 µm thickness were stained using hematoxylin–eosin (H&E) and Gram stains. Histological evaluation of these sections was performed using a light microscope (Axio Imager M1, AxioCam HRc camera; Carl Zeiss, Germany).

### Myeloperoxidase (MPO) immunohistochemistry

Paraffin-embedded tissue sections (4 µm) were dewaxed with xylene and ethanol. Sections were rehydrated in PBS (pH 7.4) for 10 min. The slides were then subjected to heat induced antigen retrieval in a decloaking chamber (Biocare Medical, CA, USA) at 110 °C for 3 min. This was followed by cooling the slides in PBS. Slides were blocked with 10% fetal bovine serum (Biological Industries, Israel, 04-001-1A), 10% bovine serum albumin (Amresco, 0332-TAM-50G) and 1% Triton × 20 (J.T.Baker,187-25-004) in PBS for 60 min. Sections were carefully dried and incubated overnight at 4 °C with polyclonal rabbit anti-human Myeloperoxidase ready to use antibody (Dako Autostainer, Ref IS511). After being washed three times in PBS, sections were incubated for 1 h with CY5-conjugated goat anti-rabbit IgG (Abcam, Cat. No. ab97077) 1:250 in PBS at room temperature. Slides were washed three times in PBS and counterstained for 20 min with 0.1 mM Sytox orange reconstituted in DMSO (Thermo Fisher, Cat. No. S11368). Slides were washed in PBS, dried off, and covered with a coverslip using an antifade media (Fluoro-Gel with Tris buffer; Cat. No. 17985-10, Electron Microscopy Sciences). Slides were examined using the Axio Imager M1 fluorescence microscope using MR3 camera (Carl Zeiss, Germany) with 50 Cy 5 and 43 HE DsRed channels.

### Statistical analysis

Statistical analyses were performed using Statistix 8 software (Analytical Software, Tallahassee, FL USA); plots were produced by Prism 5.01 (GraphPad Software; San-Diego, CA, USA). Rarefaction curves of individual samples were plotted using the vegan v2.4-2 package in R software [45]. Descriptive statistics were calculated for all swab sampled cows (*n* = 30), and for the two groups—healthy (*n* = 19) and metritis (*n* = 11) cows, according to the clinical status definition, as described above. Differences between groups in cow parity, DIM on day of sampling, percent of PMN in cytological analysis and abundance of bacterial phyla/genera were analyzed using Wilcoxon Rank Sum Test. Differences were considered significant at *P* < 0.05. Unless otherwise noted, results are presented as mean ± SEM.

For 16s rDNA sequencing, 35 cow samples were used, including swab samples (healthy *n* = 19; metritis *n* = 11) and biopsy samples (*n* = 5; healthy *n* = 2, metritis *n* = 2; septic metritis *n* = 1). As the number of reads from healthy biopsy cows was very low, 33 samples (healthy swab *n* = 19; metritis swab *n* = 11; metritis biopsy *n* = 3) were used for phylum level stacked bar plots (Figure 3), genus level MRPP test and NMDS ordination (see below). All other bacterial community analyses were performed on the swab samples (*n* = 30) exclusively. PC-ORD v6.08 (MjM Software, Gleneden Beach, OR) was used for multivariate analysis of sequencing data with Sorensen distances. For each of the swab sample groups, richness was defined as the number of OTU rows with a value greater the zero. Shannon diversity index (H) was calculated by the equation H = Σ *p*_*i*_ *ln*(*p*_*i*_), where *p*_*i*_ is the proportion of an *i*th OTU in the data matrix. Evenness (E) was calculated as E = H/ln (richness).

Differences among the swabs of healthy and metritis groups and the biopsy metritis group (reads from intercaruncular biopsy sample) were calculated by multi-response permutation procedure (MRPP) [46]. The size of the difference between the groups is represented by the A-statistic, and significance is represented by the MRPP *P*-value. Ordinations of the bacterial community of each cow were calculated by non-metric multidimensional scaling (NMDS) at 500 iterations [47].

Relative abundance was calculated as a percentage, by dividing the number of copies of a specific OTU (for some of the genera) or a set of OTUs (for Phyla, some genera) by the total number of OTUs found in either a specific cow (abundance) or a group of cows (mean abundance). Prevalence was calculated by dividing the number of cows in a specific group found to have at least one copy of the OTU of interest by the total number of cows in the group. Relative prevalence was calculated by dividing the prevalence of a specific genus in the metritis group by the prevalence of the same genus in the healthy group.

Indicator values (IV), representing the contribution of each OTU to the difference between the swab sample groups (healthy and metritis), were calculated using the method described by Dufrene and Legendre [48], based on a combination of the abundance and frequency of occurrence of each OTU in each group. The larger the IV (range 0–100), the more abundant and/or more frequent is an OTU in a given group compared to the other group; e.g., an OTU with the maximum value of 100 associated with healthy cows means the OTU is abundant and appears in all healthy cows and in none of the metritic cows.

## Results

### Clinical and endometrial cytology analyses

Overall, parity of sampled cows ranged from 1 to 6 (2.7 ± 0.3), and DIM at the sampling day from 5 to 10 (6.5 ± 0.3 days), with no significant difference between groups (healthy:parity 2.8 ± 0.4, DIM 6.8 ± 0.3 days; metritis:parity 2.4 ± 0.4, DIM 6.1 ± 0.4 days). The PMN percentage in endometrial cytology was significantly higher in cows with metritis (healthy: 10.1 ± 3.1%; metritis: 40.1 ± 13.4%; *P* = 0.024). Reproductive performance parameters were better in the healthy cows as compared to cows diagnosed with metritis (Additional files 1 and 2).

### Histopathological analysis of uterine biopsies

**Figure 1:**
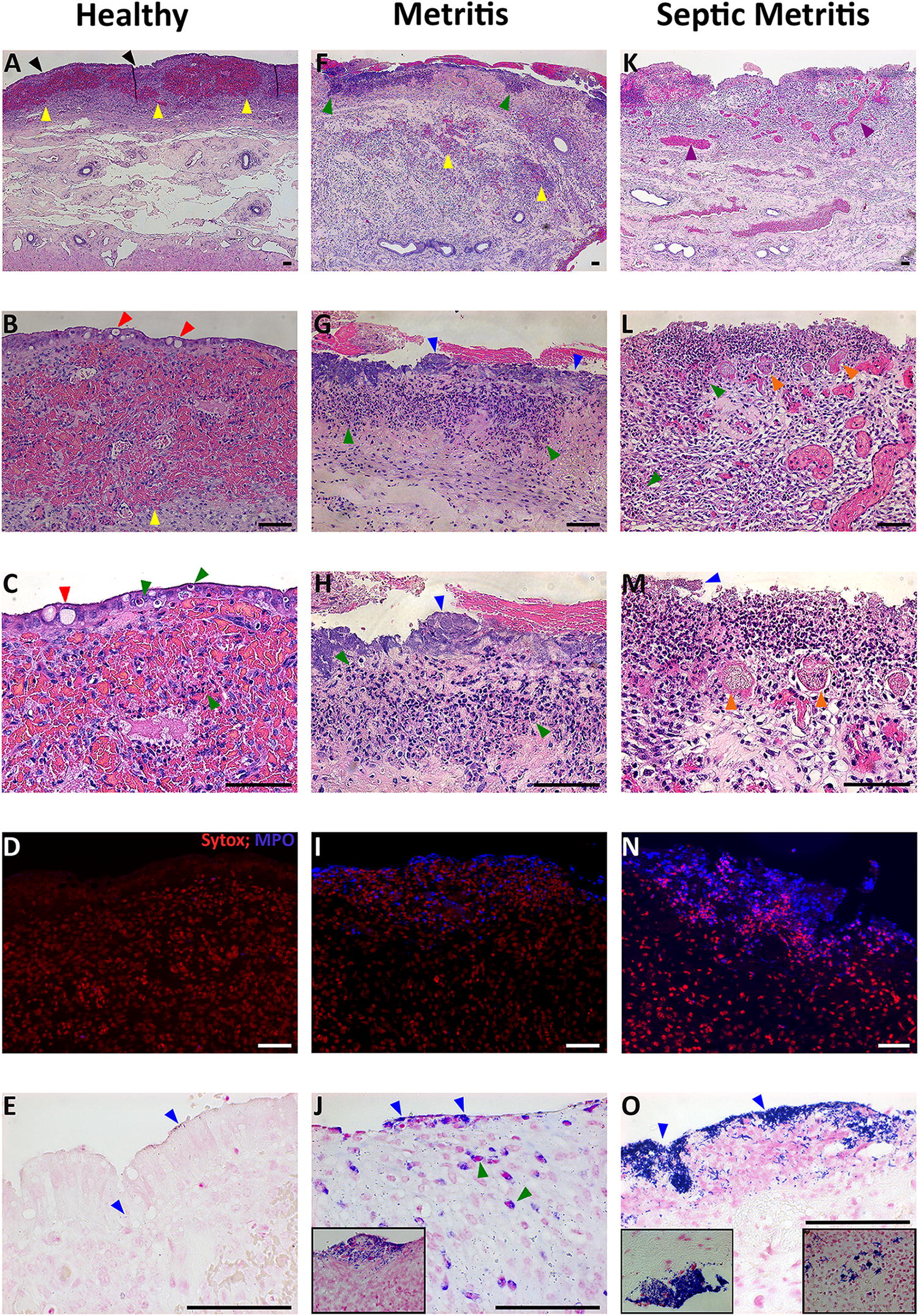
**Histopathology of the endometrium in healthy, metritic and septic metritic cows at 5–10** **days post-partum.** Scale bars in all images are 50 µm. Colors mentioned in brackets refer to the arrowheads. Healthy cows: **A**–**C** H&E stained sections, showing intact epithelium layer (black) with vacuoles (red) and PMN infiltration between cells and in the endometrium (green). A continuous hemorrhage line (yellow) can be seen adjacent to luminal epithelium. **D** Blue MPO immunohistochemistry with red Sytox orange counterstain shows minimal PMN presence. **E** Gram stain shows small amounts of bacteria (blue) on epithelium and minimal tissue invasion. Metritic cows: **F**–**H** H&E stain shows that the luminal epithelium is missing. Hemorrhages are deeper and multifocal (yellow). Foci of PMN infiltration are seen (green). Clumps of bacteria can also be seen (blue). **I** MPO immunohistochemistry shows PMN infiltration. **J** Gram stain shows clumps of bacteria on the luminal side (blue, inset), as well as phagocytosis of bacteria by PMN (green). Septic metritic cow: **K**–**M** H&E stained sections show absence of luminal epithelium. Blood vessel congestion (purple) and thrombosis (orange) are widespread, as is the massive PMN infiltration (green) and luminal bacteria presence (blue). **N** MPO immunohistochemistry confirms massive PMN infiltration. **O** Gram stain shows heavy bacterial load (blue) on luminal side, serosa (left inset) and invasion of endometrium (right inset).

The results described below are from 5 cows (healthy = 2, metritis = 2, septic metritis = 1), and as such are descriptive in nature. Histopathological evaluation of the H&E stained sections of the uterus of healthy cows revealed that most of the luminal side of the intercaruncular regions was covered by an intact single layer columnar epithelium (Figures 1A–C). Vacuoles were apparent in epithelial cells, displacing the nucleus in some cells (Figures 1B and C). PMN infiltration between epithelial cells were noted in healthy animals (Figures 1B and C). Noticeably, in both metritic and septic metritic animals the epithelium was missing from the entire endometrial luminal surface (Figures 1F–H, K–M). In the intercaruncular region of healthy animals, adjacent to the luminal side, the lamina propria shows an almost continuous line of hemorrhage accompanied by a mild, diffuse PMN infiltration (Figures 1A–C). In case of the metritic animals, hemorrhages were not organized in a line, but were rather smaller, multifocal, and extended deeper into the lamina propria (Figure 1F), accompanied by a diffuse PMN infiltration extending from the luminal side to the level of endometrial glands, with occasional foci of heavy infiltration (Figures 1G and H). In the septic metritic animal, thrombosis and vessel congestion were common findings, the PMN infiltration was extensively pronounced and was directed towards the luminal side (Figures 1K–M). The increase in PMN presence with increasing severity of the clinical presentation was confirmed by MPO Immunohistochemistry (Figures 1D, I, N).

**Figure 2:**
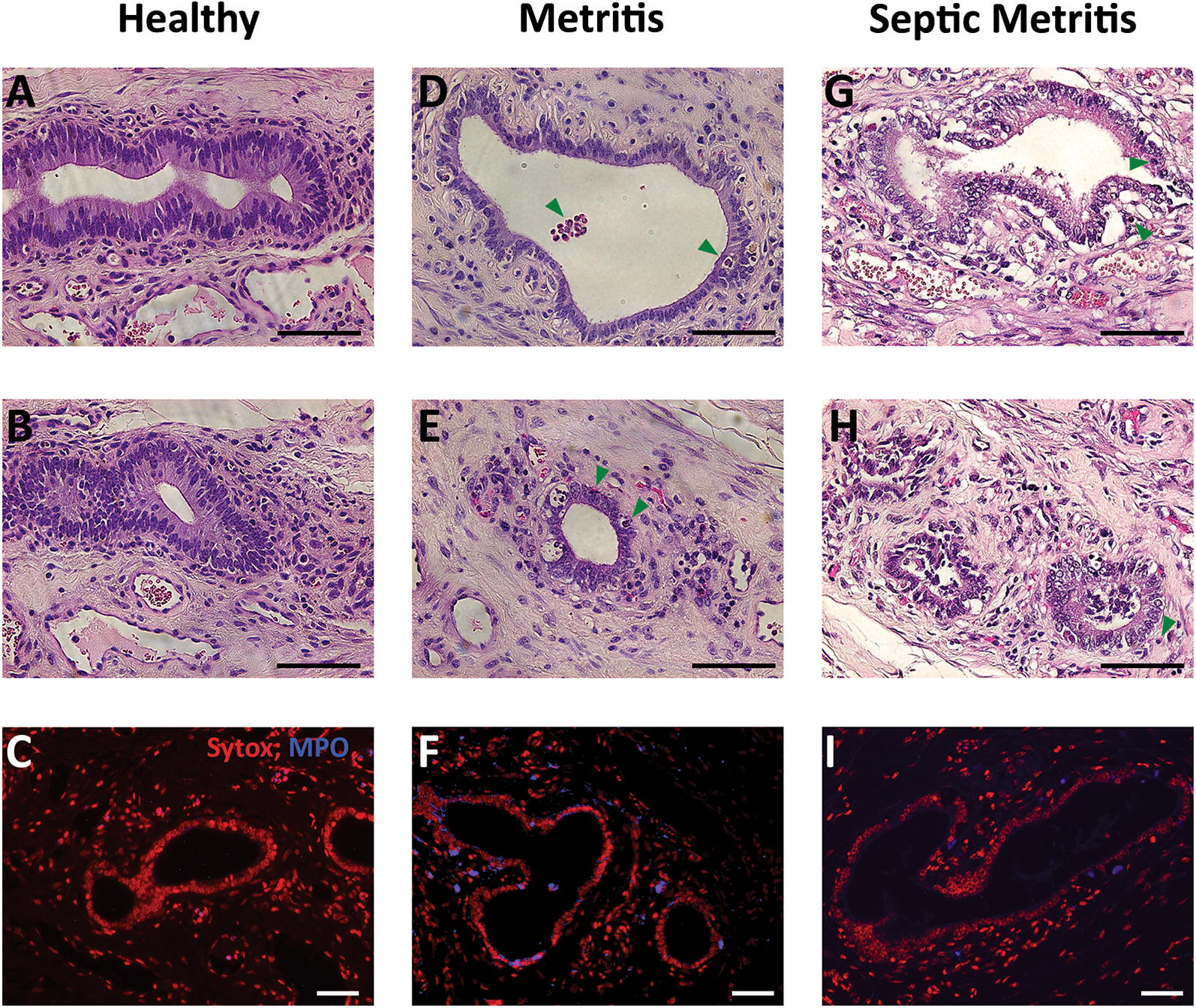
**Histopathology of endometrial glands in healthy, metritic and septic metritic cows at 5–10** **days post-partum.** Scale bars in all images are 50 µm. Healthy cows: **A**, **B** H&E stained sections, showing well preserved endometrial glands with columnar epithelium. **C** Blue MPO immunohistochemistry with red Sytox orange counterstain shows PMN presence mainly in blood vessels. Metritic cows: **D**, **E** H&E stained sections show preserved endometrial glands. Epithelial cell morphology is predominantly cuboidal. Infiltrating PMN (green arrowheads) are commonly seen in the epithelial layer and in the gland lumen. **F** MPO immunohistochemistry shows abundant PMN infiltration. Septic metritic cow: **G**, **H** H&E stains show endometrial glands in advanced stages of destruction. PMN infiltration (green arrowheads) can be seen in the epithelial layer and around the glands. **I** MPO immunohistochemistry shows PMN infiltration to a lesser extent compared to the metritis cows (F).

The endometrial glands were conserved across the uterine sections of healthy and metritic cows (Figures 2A, B, D, E). Albeit, epithelial tubular cells had a columnar appearance in healthy cows and a more cuboidal shape in metritic animals. In the septic metritic sections, glands were in advanced stages of destruction (Figures 2G and H). PMN infiltration between and into the gland lumen was more pronounced in sections from metritic and septic metritic animals (Figures 2D, E, G, H). MPO staining revealed minimal staining around the glands in healthy animals, and mild MPO staining around some of the glands in metritic cows (Figures 2C and F). In septic metritic sections there was similar or lower MPO staining observed in the deeper glands as compared to sections of metritis (Figure 2I), although PMN infiltration in the H&E histologic sections was higher in septic metritis.

Bacteria spatial distribution and load, assessed by Gram and H&E stains, were associated with disease severity. In healthy cows, bacteria were scarce and concentrated in small groups on the endometrial luminal surface of the epithelium layer, with mild penetration into the tissue (Figure 1E). In metritic animals, bacteria were concentrated in large clumps on the luminal side (Figures 1G, H, J); invasion of bacteria into the endometrium was more pronounced, accompanied by phagocytosis of bacteria by PMN (Figure 1J). In the septic metritic animal, bacteria clumps were spread across the entire luminal surface (Figures 1L, M, O), and also seen invading deeper into the endometrium as well as on the serosa surface (Figure 1O, insets). Bacteria were not clearly demonstrated in glands in the Gram stain sections.

Overall, in each cow, there were no notable differences in the H&E sections observed between intercaruncular and caruncular sections, with the exception of the expected absence of endometrial glands in the caruncular sections, and luminal epithelium in the healthy cows, as described above.

## 16S-rDNA analysis of bacterial communities in swab and biopsy samples

The 96 988 high quality sequences obtained from 30 endometrial swab samples were assigned to 969 genus-level OTUs. Average number of reads per cow was 3233 ± 393 reads. Rarefaction curves (Additional file 3) reached a plateau for most cows, indicating that the sequences obtained closely represent the bacterial diversity of the sample taken from each cow [49]. Mean richness, evenness and Shannon diversity index of the healthy group were 54.6 ± 21.1, 0.4 ± 0.06 and 1.5 ± 0.3, respectively; in the metritis group, the parallel values were 45.5 ± 5.7, 0.4 ± 0.03 and 1.5 ± 0.2, respectively. None of the three alpha diversity parameters were significantly different between the groups.

**Figure 3:**
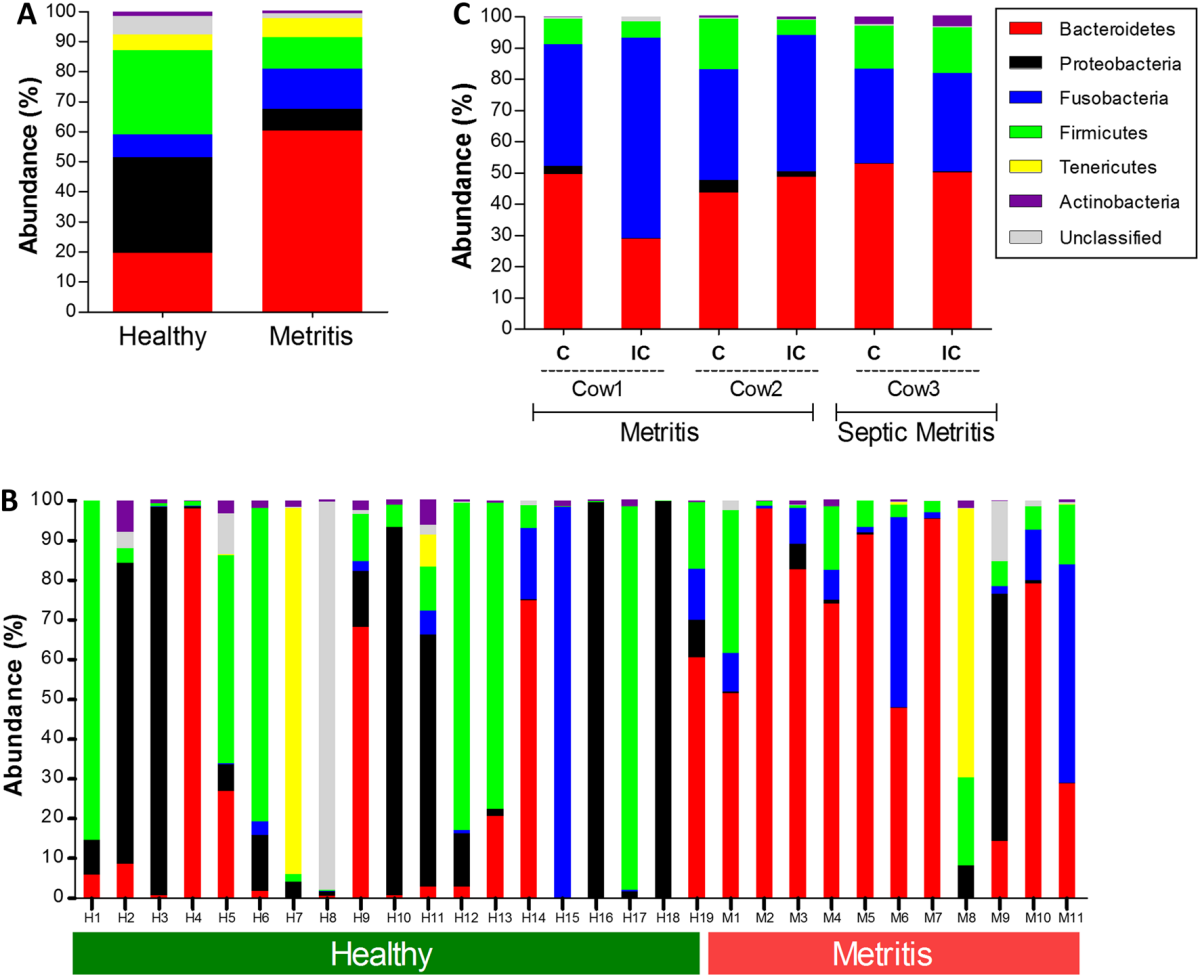
**Stacked-bar plots of endometrial bacterial community composition by phylum, in healthy, metritic and septic metritic cows. A** Average community composition of swab samples by phylum in healthy cows (*n* = 19) as compared to cows diagnosed with metritis (*n* = 11). Note the increased abundance of the Bacteroidetes phylum in metritic cows, mainly at the expanse of the Proteobacteria and Frimicutes phyla. **B** Community composition of individual endometrial swab samples by phylum in healthy cows (*n* = 19) and cows diagnosed with metritis (*n* = 11). Note the relatively uniform bacterial community composition of metritic cows compared to the more diverse composition of healthy cows. **C** Community composition of individual uterine biopsy samples from cows diagnosed with metritis (*n* = 2 cows) or septic metritis (*n* = 1 cow); C: caruncular samples; IC: inter-caruncular sample. Note the similarity between these abundance plots, the individual plots of metritic swab samples (B), and the mean metritic community composition (A).

For each of the swab cow groups (healthy vs. metritis), mean abundance of phyla was calculated, as summarized in Additional file 4 and presented in Figure 3A. In descending order, the most abundant phyla in healthy cows were Proteobacteria, Firmicutes and Bacteroidetes, and in metritic cows Bacteroidetes, Fusobacteria and Firmicutes. A statistically significant difference was found in the average percentage of the Bacteroidetes, Fusobacteria and Proteobacteria phyla between healthy and metritic cows. Bacterial community composition by phylum was also calculated separately for each individual swab sample (Figure 3B). The composition of the bacterial communities of healthy cows varied, and presented a wider set of options compared to the community compositions found in the metritic cows, which appear to be relatively uniform among most cows in that group. In addition, MRPP analysis showed a significant difference between the bacterial communities of healthy and metritic cows (swab metritis *A* = 0.0280, *P *= 0.018).

**Figure 4:**
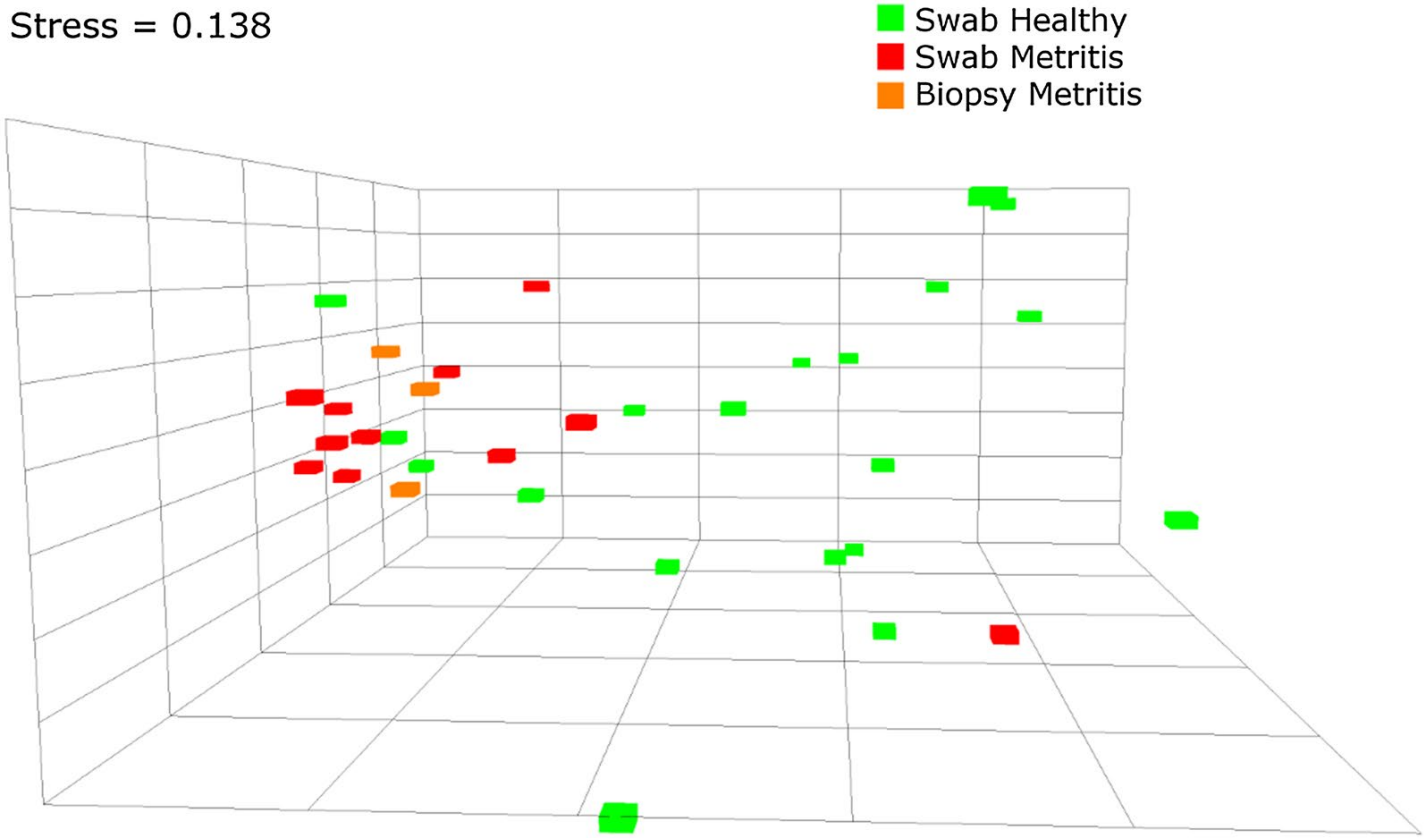
**Non-metric Multidimensional Scaling (NMDS) ordination plot of endometrial bacterial community composition, in healthy and metritic cows.** The axes and dimensions in NMDS are arbitrary. The “stress” value is a goodness of fit measure, representing how well the low dimensionality (3D) ordination represents the actual multi-dimensional (969D, as the number of OTUs) distance between each pair of samples. The lower the stress, the better the ordination. Stress values between 0.2 and 0.1 are considered acceptable. Notice the clustering of metritic cows (red and orange), in contrast to the more dispersed population of healthy cows (green). The biopsy metritic cows seem to be a subgroup of the swab metritis group, perhaps owing to differential penetration of bacteria from the lumen and epithelial surface (swab samples) to the interior structures of the uterine wall (biopsy samples).

High quality read numbers from biopsy samples are summarized in Table 1. All healthy cow biopsy samples produced low read numbers, which prevented us from mapping the community composition in these cows. Stacked bar plots of community composition by phyla were constructed for each of the metritic cow biopsy samples, as shown in Figure 3C. MRPP analysis of biopsy samples compared to healthy swab samples showed a significant difference between the bacterial communities (*A* = 0.0313, *P* = 0.032). There was also a tendency for a difference between the swabs and biopsies of metritic cows (*A* = 0.0496, *P* = 0.061), which could be explained by differences in sample size, technical differences, or by the biopsy metritis group being a subset of the swab metritis group, with a much smaller intra-group distance, as is visible in the NMDS plot (Figure 4).

**Table 1:** **High-quality read numbers of uterine biopsy samples collected from healthy, metritic and septic metritic cows**

Tissue	Healthy		Metritis		Septic metritis
	Cow 1	Cow 2	Cow 3	Cow 4	Cow 5
Caruncle	1	20	5093	1494	2476
Inter-caruncle	2	17	1959	4187	2203

Number of reads obtained from 16S-rDNA pyrosequencing of biopsy samples, after trimming, denoising and quality control.

NMDS analysis (3D stress value = 0.138) of healthy and both metritic groups (swab and biopsy samples) shows the same pattern as the individual stack bar plots (Figure 3B)—the bacterial communities of healthy cows are more dispersed along the axes, while the metritic group communities tend to cluster together. The metritis biopsy samples seem to represent a subgroup of the swab metritis group.

Prevalence and relative abundance of genera that are presumed to be metritis-associated pathogens according to previous studies were calculated for the swab samples (Table 2). *Escherichia* and *Shigella* genera have a high similarity in the V1–V3 region of the 16S-rRNA gene and are thus classified as a combined OTU (*Escherichia/Shigella*) according to the methods used in this study.

**Table 2:** **Prevalence and mean abundance (± SEM) of selected bacterial genera in healthy and metritic cows**

Genus	Prevalence		Abundance		*P* value
	Healthy (%)	Metritis (%)	Healthy (%)	Metritis (%)	
*Bacteroides*	42.1	27.3	4.3 ± 2.0	10.7 ± 4.4	0.121
*Escherichia*/*Shigella*^a^	26.3	9.1	10.8 ± 6.2	0.5 ± 0.5	0.261
*Fusobacterium*	68.4	90.9	2.8 ± 1.2	12.4 ± 5.2	0.013
*Porphyromonas*	47.4	90.9	10.2 ± 4.9	29.8 ± 8.0	0.011
*Trueperella*	47.4	72.7	0.2 ± 0.1	0.2 ± 0.1	0.288

Samples were collected by endometrial swabs at 5–10 days post-partum and used for 16S-rDNA pyrosequencing analysis.

^a^The *Escherichia/Shigella* genera have a high similarity in the 16S-rRNA gene and thus both appear as the same OTUs according to the protocols used in this study.

**Figure 5:**
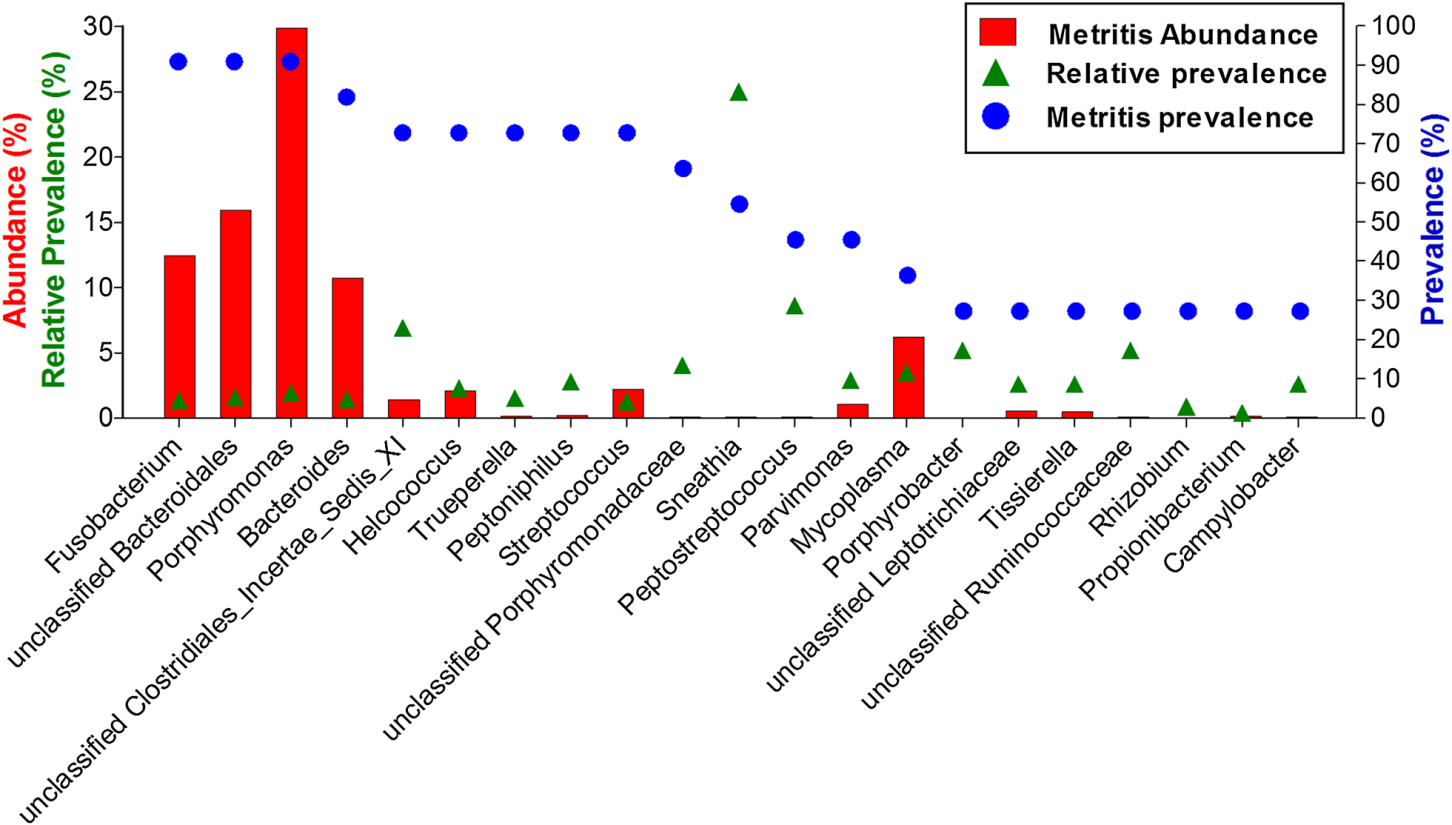
**Prevalence, abundance and relative prevalence of frequent bacterial genera in metritic cows.** Right Y axis: the prevalence of genera that were present in >25% of the metritic cows is shown by the blue dots. Left Y axis: the red bars represent the mean abundance of the genera in metritic cows. The relative prevalence (prevalence of a genus in metritic cows divided by the prevalence of the same genus in healthy cows) is shown by the green triangles. Where the denominator (prevalence in healthy cows) was zero (i.e., *Sneathia*) an arbitrary value of 25 was assigned.

Bacterial genera associated with clinical status (healthy or metritis) with indicator values (IV) above 50 are shown in Table 3. Relative prevalence was calculated for the most prevalent genera in the metritis group (i.e., present in >25% of metritic cows), by dividing the prevalence of a genus in the metritis group by the prevalence in the healthy group (Figure 5). This analysis showed a number of genera which present a combination of low abundance and high relative prevalence, such as *Sneathia*, *Peptostreptococcus* and *Porphyrobacter*. The genus *Sneathia* (phylum Fusobacteria) is outstanding in this aspect, with a prevalence of 0% in the healthy group compared to 54.5% in the metritis group (since the denominator is zero, an arbitrary value of 25 was assigned for the relative prevalence of *Sneathia*).

**Table 3:** **Indicator values of statistically significant genera in relation to clinical status (healthy vs. metritis) in post-partum cows**

Genus	Phylum	Clinical status	Indicator value	*P* value
*Fusobacterium*	Fusobacteria	Metritis	74	0.019
*Propionibacterium*	Actinobacteria	Healthy	65.4	0.011
*Bacteroides*	Bacteroidetes	Metritis	63.4	0.002
*Acinetobacter*	Proteobacteria	Healthy	60.8	0.012
*Cupriavidus*	Proteobacteria	Healthy	60.7	0.011
*Porphyromonas*	Bacteroidetes	Metritis	52.9	0.048

Indicator value (IV) is an asymmetric index [48], used in this study to associate bacterial genera to cow clinical status, based on abundance and prevalence. The more abundant and prevalent the genus is in one group with a defined clinical status compared to the other, the higher the IV.

Some healthy cows (H4, H9, H14 and H19) present a bacterial community composition that is somewhat similar to the average metritic cow composition when classified by phyla. These four cows were designated as a subgroup, and analysis of the most abundant genera of each of the three dominant phyla (Bactroidetes, Firmicutes, Fusobacteria) were compared to the metritis group; as summarized in Additional file 5, there was no significant difference between the two subgroups average abundances in that analysis. Furthermore, these healthy cows were close to the metritis group in the NMDS ordination plot (labels not shown). However, the most notable difference was in the Fusobacteria phylum, with the genera *Streptobacillus* and *Sneathia* present only in the metritic cows but absent in the subgroup of these four healthy cows.

## Discussion

The results of this study emphasize the differences between healthy Holstein–Friesian dairy cows to cows diagnosed with metritis in the very early lactation, in regards to their endometrial inflammation (as shown by cytology and histology analyses), bacterial communities composition, spatial distribution and load (as shown by the 16S-rDNA analysis and histopathology), as well as their reproductive performance. In Israel, most dairy cows are routinely examined by their veterinarian in order to clinically diagnose metritis according to the criteria described in this study. Endometrial cytological analysis during the first 10 days post-partum is not commonly performed. Interestingly, in this study, cows with metritis had a significantly higher cytologic PMN percentage at 5–10 DIM as compared to healthy cows; which is somewhat parallel to the results of other studies comparing cows with and without endometritis, commonly diagnosed cytologically much later in lactation [50, 51]. The presence of a relatively high PMN percentage in cytological samples from healthy cows can be attributed to the early stages of the uterine involution process, which involves massive tissue remodeling and replacement, mediated by an inflammatory process, or possibly due to low sensitivity of the clinical classification. Another possible explanation for this finding is the presence of PMN as part of the innate immune reaction to bacterial contamination of the uterine lumen. The lower percentage of PMN in healthy cows might be due to an appropriate response to infection or greater tissue damage in metritic cows. However, it can also suggest a controlled and efficient inflammatory reaction in healthy cows, in contrast to excessive PMN mobilization to the uterus in metritic cows associated with the relevant clinical signs. This finding is concurrent with the difference in the severity and depth of infiltration and in PMN mobilization observed between healthy and metritic cows in the histopathologic analysis, suggesting that cytological analysis of the superficial layer of the endometrium and luminal fluid can closely indicate the severity of the inflammatory process in deeper layers of the uterus [29]. Better reproductive performance of the healthy cows compared to cows diagnosed with metritis at 5–10 DIM may emphasize the potential benefit of such an early examination and diagnosis, as has been previously demonstrated by Goshen and Shpigel [31].

The histopathologic analysis results of the healthy cows are similar to those reported by previous studies [32–34]. The almost continuous line of hemorrhage observed adjacent to the luminal epithelium in the healthy cows led us to speculate as to the role of these hemorrhages in the early stages of normal involution. A possible explanation is that in healthy cows this continuous hemorrhage line may perhaps act as a barrier against bacteria trying to invade into the deeper layers of the uterus, either by mechanical interference (clot formation) or by concentrating the blood borne leukocytes close to the luminal surface, which serves as the entry point for invading bacteria. The ability of bacteria to invade the endometrium to a larger extent in metritic cows is probably aided by the absence of endometrial epithelial layer of these cows [52]. It is unclear from the current study whether this is a host defect acting as a risk factor for the development of metritis, or the result of the inflammatory process of metritis initiated by the presence of luminal bacteria. We used MPO as a marker for PMN presence as they contain a rich supply of the green heme enzyme MPO [53, 54]. In general, the PMN were concentrated mainly in the luminal layers of the uterus. We could not observe deeper migration of PMN into the inner layers of the uterus (muscularis, serosa) in metritic animals, but the presence of PMN in the endometrium seems to be associated with the clinical presentation. Still, the relatively small number of cows used for histopathological sections (healthy = 2 cows, metritis = 2 cows, septic metritis = 1 cow) do not allow robust statistical quantitative or semi-quantitative analysis; therefore, these results are descriptive in nature, and limit us from drawing definitive conclusions or causal inferences.

The bacterial community compositions in healthy cows exhibited a more diverse array as shown in individual stacked bar plots and NMDS analysis, while the bacterial community among cows with metritis was more uniform (as shown in Figures 3B and 4). These findings further strengthen the concept presented in previous studies [15, 21] of a limited set of pathogenic bacterial communities acting as the etiologic agents of metritis rather than few pathogens, each acting as single, independent etiologic agent.

The genus *Fusobacterium*, showed high prevalence and mean abundance in the swabs of cows in the metritic group. However, genera which are presumed to be important pathogens in the development of metritis in studies using culture-dependent methods [7], such as *Escherichia* and *Trueperella*, showed low mean abundance in the metritis group in the current study (even when assuming the *Escherichia/Shigella* OTU as representing the *Escherichia* genus only). *Porphyromonas* has been linked to bovine necrotic vulvovaginitis in a culture based study [55], but is not often present in cultures from uteri of metritic cows. Nevertheless, *Porphyromonas* is emerging as one of the most abundant pathogens associated with metritis in studies using culture-independent methods [18, 56], and appeared at higher prevalence and significantly higher mean abundance in the metritis group in this study. These findings are in line with the recent study by Cunha et al. [57], in which *Escherichia coli* and *T. pyogenes* quantification did not differ between healthy and metritic cows, while *F. necrophorum* and *Porphyromonas levii* quantification did differ between the groups. The results suggest a possible bias in studies relying on culture-dependent methods towards species amenable to cultivation. However, bias also exists in studies of microbial communities based on sequence methodologies, mainly due to choice of DNA extraction method or materials, the initial PCR amplification [58], primer selection [59], as well as sequencing and taxonomic classification errors. We would like to suggest accepting data from culture-based and culture-independent studies as complementary parts of the same picture, while taking into consideration the potential bias arising from each of these methods.

Indicator values calculated in this study showed *Bacteroides*, *Fusobacterium* and *Porphyromonas* to be associated with metritis, supporting other type of analyses performed in this and other studies [56, 57, 60]. *Acinetobacter*, *Cupriavidus* and *Propionibacterium* were associated with healthy cows; these three genera belong to phyla more abundant in healthy cows—Proteobacteria and Actinobacteria.

The bacterial community analyses discussed above are based at least in part on abundance. In order to further explore the components of the microbial communities, the relative prevalence of high and low abundance genera were calculated. Interestingly, this analysis revealed high relative prevalence of certain low abundance genera in the metritis group, such as *Peptostreptococcus* and *Sneathia*. *Peptostreptococcus* has already been suggested to be involved in uterine infections [18, 21]. Association of *Sneathia* with metritis in this study is in contrast to the findings reported in a recent study [21] which associated *Sneathia* with uterine health. Assuming a different environmental bacteria in the different studies (Florida vs. Israel), these conflicting results could be attributed to the contribution of the environmental bacteria in each study to the development of bacterial communities of the post-partum uterus. These high relative prevalence bacterial genera might have a larger role in metritis development than suggested by their relative abundance.

The similarity between the mean community composition of swab and biopsy samples in metritic cows could indicate that the main components of the bacterial community present on the surface epithelium and in the lumen (swab sample) are able to penetrate into the uterine tissue (biopsy sample); this is supported by the higher degree of bacterial invasion in metritic cows demonstrated in the Gram stained sections. However, differences are still present, in accordance with the recent report by Knudsen et al. [20]. Nevertheless, the biopsy samples from the metritic cows seem to form a subgroup of the swab metritic communities. This observation can be explained by the different tissue penetrating abilities certain bacteria possess, by which only some members of the metritic bacterial communities found in the lumen and barrier epithelium can actually invade the uterine wall and cause disease [20, 61, 62].

One of the open questions regarding the pathophysiology of metritis is whether the ability of components of the bacterial communities to penetrate and infect the uterus is the primary cause of metritis, or alternatively, are certain cows unable to mount an effective immune response to a pathogenic bacterial community in the uterine lumen and therefore develop metritis, resulting in barrier dysfunction (such as epithelial necrosis, inadequate immune response), thus allowing the bacteria to invade the uterus. This question further resonates in our finding that some healthy cows present a community composition resembling that of metritic cows; however, there were still notable differences in the Fusobacteria phylum, with the genera *Streptobacillus* and *Sneathia* present only in the metritic cows but absent in the subgroup of these healthy cows.

The low number of high quality reads in the biopsy samples from healthy cows could be explained by technical errors, artefacts, a low ratio of bacterial DNA to bovine DNA (which composes most of the DNA extracted from the biopsy samples), sequencing error, or due to low sample size. However, if we assume that the low number of bacterial OTUs found in the healthy cow biopsy samples represents the true relative distribution of bacteria in the uterus tissue, a possible explanation is that while the uterine lumen of healthy cows is populated by bacterial communities (swab samples), the bacteria are not able to effectively invade into the uterus. Karstrup et al. [63] demonstrated that bacterial invasion from the lumen into the uterus is less frequent in healthy cows, and this can also be seen in the Gram stains in this study. Unfortunately, as taking full thickness biopsies is a complicated and invasive procedure, the amount of tissue samples in this study was limited and cannot allow us to reach binding conclusions as to the role of bacterial communities in swabs as compared to biopsies in healthy and metritic cows.

In summary, the current study indicates the differences between healthy Holstein–Friesian dairy cows and cows diagnosed with metritis at 5–10 days post-partum, in regards to the characteristics of the endometrial inflammation and bacterial communities’ composition, spatial distribution and load, as well as the consequences on their reproductive performance. Bacterial community composition analysis revealed a unique bacterial community in most cows suffering from metritis, as compared to more diverse communities among cows which were defined as healthy. The inflammatory reaction and the luminal epithelium integrity differ between healthy cows and cows with metritis. Although yet to be fully understood, it is clear that the interplay between bacterial community composition and host response and defense mechanisms is important to disease development, as supported by the findings in the current study in regards to the epithelial layer integrity, PMN infiltration, localization and activity, in healthy cows compared to metritic cows. As post-partum uterine inflammatory diseases are among the most prevalent and economically impacting diseases in bovine dairy herds worldwide, understanding the pathophysiology of metritis is critical for developing effective therapies and prevention means in order to improve reproductive performance and animal welfare.

## Additional files

**Additional file 1.** **Reproductive performance of healthy and metritic cows.** Reproductive management was based on artificial insemination (AI) with thawed-frozen semen from proven sires. AI was performed by highly trained technicians employed by Sion LTD., Israel’s leading company for dairy cow Artificial Insemination Service. Cows were bred on spontaneous estrus observed or detected by computerized pedometry system. Estrus was confirmed by trans-rectal palpation of the reproductive tract at the time of AI. For pregnancy diagnosis, trans-rectal palpation of the uterus was performed at 40–50 days post-insemination. Reproductive performance parameters included the following: number of AI to conception, open days (days from parturition to conception), waste days (days from first AI to AI leading to conception; i.e., equals 0 if cow is pregnant from first AI), and pregnancy rate at 180 DIM. Data were analyzed by Wilcoxon rank sum test, or a Pearson’s Chi square test analysis (to compare pregnancy rate at 180 DIM). Values presented in the table are mean ± SEM for each group, or percentages (%).

**Additional file 2.** **Kaplan–Meyer survival analysis of time interval from parturition to pregnancy in healthy and metritic cows.** Kaplan-Meier Survival analysis (Log Rank, Mantel-Cox) test was used to analyze differences in interval from parturition to conception. Time to pregnancy was significantly shorter in healthy cows (green) as compared to cows with metritis (red), *P* = 0.004.

**Additional file 3.** **Rarefaction curves of 16S-rDNA pyrosequencing endometrial samples from healthy, metritic and septic metritic cows.** Rarefaction curves of the number of OTUs are presented as a function of read number. H - healthy cows, endometrial swab samples; M - metritic cows, endometrial swab samples; BM - metritic cows, full-thickness uterine biopsy samples; BS - septic metritis cow, full-thickness uterine biopsy sample.

**Additional file 4.** **Endometrial bacterial community composition by phylum in healthy and metritic cows.** Samples were collected by endometrial swabs at 5–10 days post-partum and used for 16S-rDNA pyrosequencing analysis. Results are presented as mean abundance ± SEM. *P* value is specified where a statistically significant difference was found between the groups; NS: not significant.

**Additional file 5.** **Most abundant genera of the phyla Bacteroidetes, Firmicutes and Fusobacteria in healthy and metritic cows with similar community composition.** Samples were collected by endometrial swabs at 5–10 days post-partum and used for 16S-rDNA pyrosequencing analysis. For each phylum, the ten most abundant OTUs were included in descending order. Some genera appear twice or more as distinct OTUs. *The healthy group is composed of four cows with a similar bacterial community composition to the average metritic cow community composition sorted by phylum.

## Abbreviations

AI: artificial insemination
DIM: days in milk
H&E: hematoxylin–eosin
IV: indicator value
MPO: myeloperoxidase
MRPP: multi-response permutation procedure
NMDS: non-metric multidimensional scaling
OTU: operational taxonomic unit,
PBS: phosphate buffered saline
PMN: polymorphonuclear cells
