# Supplementary material for: Microbial communities and inflammatory response in the endometrium differ between normal and metritic dairy cows at 5–10 days post-partum
Source: Vet Res. 2018 Aug 2;49:77. doi: 10.1186/s13567-018-0570-6 (PMC6071394; doi:10.1186/s13567-018-0570-6)
Supplement: Supplementary file 1 — Additional file 1. Reproductive performance of healthy and metritic cows. Reproductive management was based on artificial insemination (AI) with thawed-frozen semen from proven sires. AI was performed by highly trained technicians employed by Sion LTD., Israel’s leading company for dairy cow Artificial Insemination Service. Cows were bred on spontaneous estrus observed or detected by computerized pedometry system. Estrus was confirmed by trans-rectal palpation of the reproductive tract at the time of AI. For pregnancy diagnosis, trans-rectal palpation of the uterus was performed at 40–50 days post-insemination. Reproductive performance parameters included the following: number of AI to conception, open days (days from parturition to conception), waste days (days from first AI to AI leading to conception; i.e., equals 0 if cow is pregnant from first AI), and pregnancy rate at 180 DIM. Data were analyzed by Wilcoxon rank sum test, or a Pearson’s Chi square test analysis (to compare pregnancy rate at 180 DIM). Values presented in the table are mean ± SEM for each group, or percentages (%). [file 13567_2018_570_MOESM1_ESM.docx]

|  | **Healthy** | **Metritis** | ***P* value** |
| --- | --- | --- | --- |
| **# AI to conception** | 1.7 ± 0.2 | 2.8 ± 0.8 | 0.255 |
| **Open Days** | 113.3 ± 7.2d | 166 ± 21.9d | 0.021 |
| **Waste Days** | 21.5 ± 5.3d | 63.2 ± 20.8d | 0.027 |
| **Pregnancy rate at 180 DIM** | 87.5% | 50% | 0.036 |
