# Supplementary figures and images for: Microbial communities and inflammatory response in the endometrium differ between normal and metritic dairy cows at 5–10 days post-partum
Source: Vet Res. 2018 Aug 2;49:77. doi: 10.1186/s13567-018-0570-6 (PMC6071394; doi:10.1186/s13567-018-0570-6)

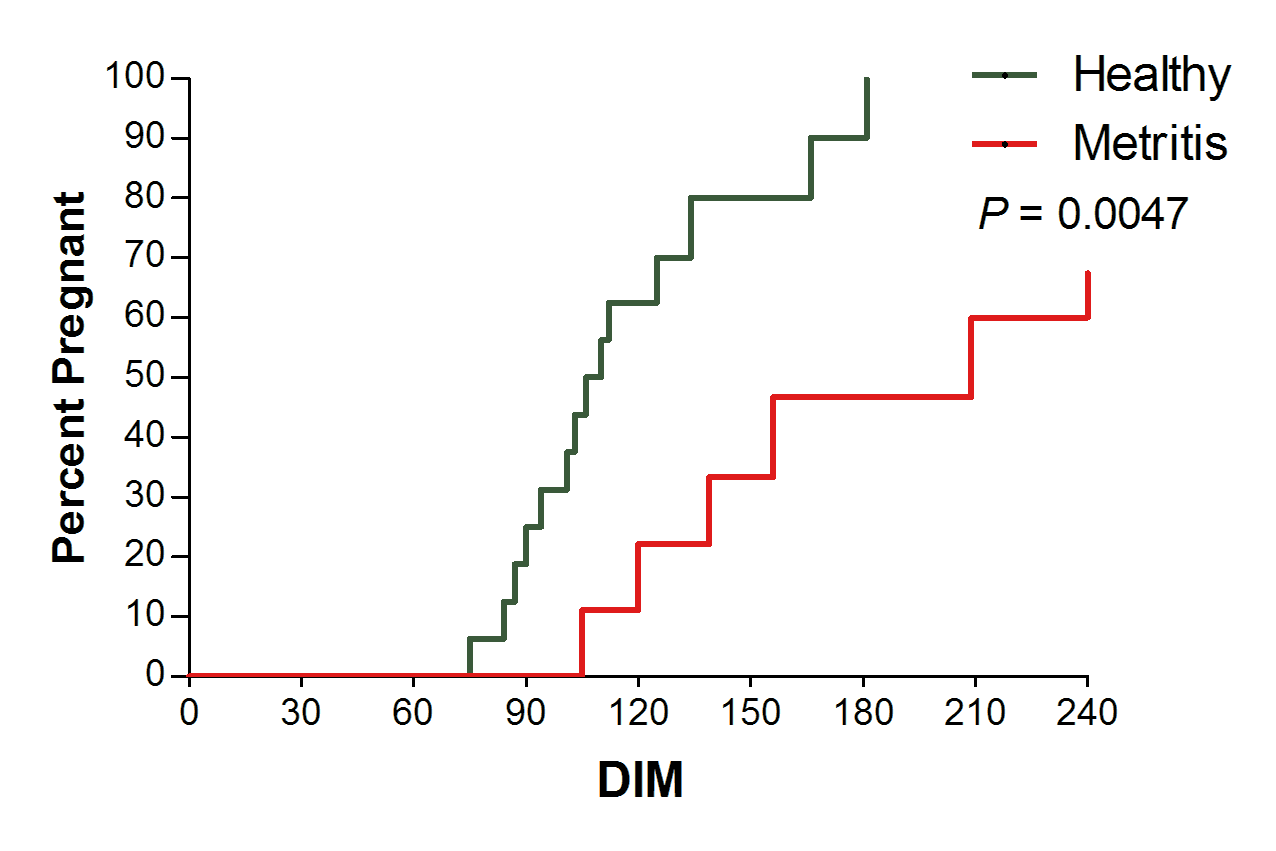

Supplement: Supplementary file 2 — Additional file 2. Kaplan–Meyer survival analysis of time interval from parturition to pregnancy in healthy and metritic cows. Kaplan-Meier Survival analysis (Log Rank, Mantel-Cox) test was used to analyze differences in interval from parturition to conception. Time to pregnancy was significantly shorter in healthy cows (green) as compared to cows with metritis (red), P = 0.004. [file 13567_2018_570_MOESM2_ESM.tif]

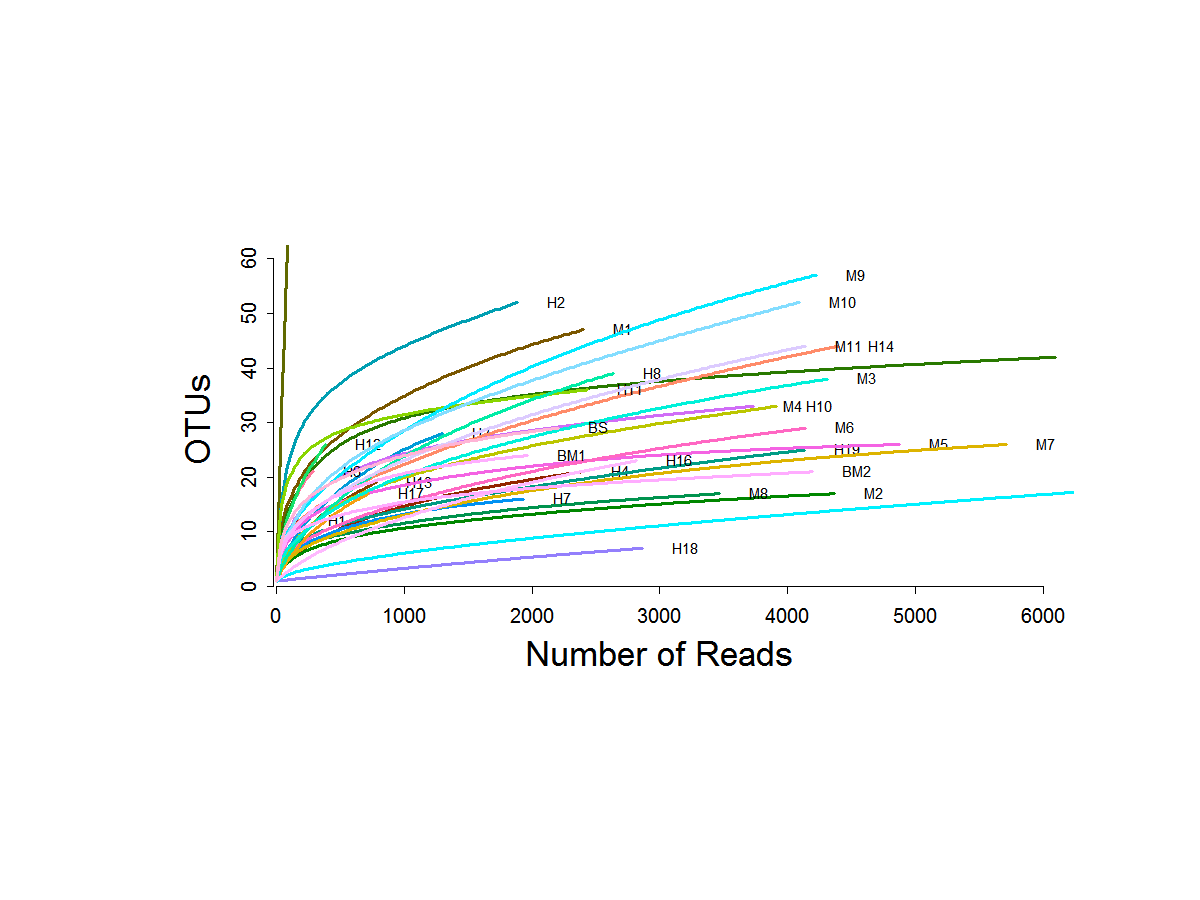

Supplement: Supplementary file 3 — Additional file 3. Rarefaction curves of 16S-rDNA pyrosequencing endometrial samples from healthy, metritic and septic metritic cows. Rarefaction curves of the number of OTUs are presented as a function of read number. H - healthy cows, endometrial swab samples; M - metritic cows, endometrial swab samples; BM - metritic cows, full-thickness uterine biopsy samples; BS - septic metritis cow, full-thickness uterine biopsy sample. [file 13567_2018_570_MOESM3_ESM.tiff]
