# Supplementary material for: Microbial communities and inflammatory response in the endometrium differ between normal and metritic dairy cows at 5–10 days post-partum
Source: Vet Res. 2018 Aug 2;49:77. doi: 10.1186/s13567-018-0570-6 (PMC6071394; doi:10.1186/s13567-018-0570-6)
Supplement: Supplementary file 4 — Additional file 4. Endometrial bacterial community composition by phylum in healthy and metritic cows. Samples were collected by endometrial swabs at 5–10 days post-partum and used for 16S-rDNA pyrosequencing analysis. Results are presented as mean abundance ± SEM. P value is specified where a statistically significant difference was found between the groups; NS: not significant. [file 13567_2018_570_MOESM4_ESM.docx]

| ***P* value** | **Metritis (%)** | **Healthy (%)** | **Phylum** |
| --- | --- | --- | --- |
| NS | 0.5 ± 0.2 | 1.5 ± 0.5 | **Actinobacteria** |
| 0.004 | 60.3 ± 10.3 | 19.7 ± 7.2 | **Bacteriodetes** |
| NS | 0 ± 0 | 0.04 ± 0.04 | **Deinococcus-Thermus** |
| NS | 10.5 ± 3.3 | 27.9 ± 8.4 | **Firmicutes** |
| 0.031 | 13.4 ± 5.9 | 7.5 ± 5.2 | **Fusobacteria** |
| 0.005 | 7.3 ± 5.6 | 31.8 ± 9.3 | **Proteobacteria** |
| NS | 0 ± 0 | 0.02 ± 0.02 | **Spirochetes** |
| NS | 6.3 ± 6.2 | 5.3 ± 4.8 | **Tenericutes** |
| NS | 0 ± 0 | 0.1 ± 0.1 | **Verrucomicrobia** |
| NS | 1.7 ± 1.4 | 6.1 ± 5.1 | **Unclassified** |
