# Supplementary material for: Microbial communities and inflammatory response in the endometrium differ between normal and metritic dairy cows at 5–10 days post-partum
Source: Vet Res. 2018 Aug 2;49:77. doi: 10.1186/s13567-018-0570-6 (PMC6071394; doi:10.1186/s13567-018-0570-6)
Supplement: Supplementary file 5 — Additional file 5. Most abundant genera of the phyla Bacteroidetes, Firmicutes and Fusobacteria in healthy and metritic cows with similar community composition. Samples were collected by endometrial swabs at 5–10 days post-partum and used for 16S-rDNA pyrosequencing analysis. For each phylum, the ten most abundant OTUs were included in descending order. Some genera appear twice or more as distinct OTUs. *The healthy group is composed of four cows with a similar bacterial community composition to the average metritic cow community composition sorted by phylum. [file 13567_2018_570_MOESM5_ESM.docx]

| **Phylum / Group** | **Healthy^*^** | **Metritis** |
| --- | --- | --- |
| **Bacteroidetes** | *Porphyromonas* | *Porphyromonas* |
|  | Unclassified, order *Bacteroidales* | Unclassified, order *Bacteroidales* |
|  | *Bacteroides* | *Bacteroides* |
| **Firmicutes** | *Streptococcus* | *Streptococcus* |
|  | *Helcococcus* | *Tissierella* |
|  | Unclassified, Family *Clostridiales Incerta Sedis XI* | *Helcococcus* |
|  | *Parvimonas* | *Peptoniphilus* |
|  | *Tissierella* | Unclassified, Family *Veillonellaceae* |
|  | Unclassified, order *Clostridiales* | *Parvimonas* |
|  | Unclassified, Family *Veillonellaceae* | *Finegoldia* |
| **Fusobacteria** | *Fusobacterium* | *Fusobacterium* |
|  | Unclassified, family *Leptotrichiaceae* | Unclassified, family *Leptotrichiaceae* |
|  | *streptobacillus* |  |
|  | *Sneathia* |  |
